# Cross-cultural adaptation, reliability and validity of the Turkish version of the stroke exercise preference inventory

**DOI:** 10.3389/fpsyg.2025.1535140

**Published:** 2025-02-13

**Authors:** Halime Arıkan, Meral Sertel

**Affiliations:** ^1^Department of Physiotherapy and Rehabilitation, Faculty of Health Sciences, Tokat Gaziosmanpaşa University, Tokat, Türkiye; ^2^Department of Physiotherapy and Rehabilitation, Faculty of Health Sciences, Bursa Uludağ University, Bursa, Türkiye

**Keywords:** validity, reliability, stroke, stroke exercise preference inventory, Turkish version

## Abstract

**Introduction:**

While studies on version adaptation, validity, and reliability are common, no tools exist in Turkish literature to assess exercise preferences in stroke patients. This research aimed to translate the Stroke Exercise Preference Inventory (SEPI) into Turkish and evaluate its validity and reliability in stroke patients.

**Methods:**

Ninety stroke patients completed the SEPI, Exercise Benefits/ Barriers Scale (EBBS), Behavioral Regulation in Exercise Questionnaire (BREQ-2), Stroke-Specific Quality of Life Scale (SSQoLS), and Frenchay Activities Index (FAI). The SEPI was translated into Turkish using a standard forward-backward translation process. Psychometric properties such as structural and construct validity, test–retest reliability, and internal consistency were assessed.

**Results:**

Reliability analysis demonstrated high internal consistency for SEPI-13, with Cronbach’s *α* values of 0.931. Validity testing revealed a 3-factor structure for SEPI-13, explaining 69.029% of total variance. CFA confirmed the model with acceptable fit indices. Construct validity showed good correlations with EBBS (*r* = −0.771; *p* < 0.001) and BREQ-2 (*r* = from −0.541 to 0.732; *p* < 0.001) for convergent validity, while divergent validity was supported by weak correlations with SSQoLS (*r* = 0.165; *p* = 0.120) and FAI (*r* = 0.137; *p* = 0.197). No floor or ceiling effects were observed for SEPI-13.

**Discussion:**

The Turkish SEPI is a reliable and valid tool for assessing exercise preferences in stroke patients, aiding their rehabilitation.

## 1 Introduction

Stroke is a significant concern for public health systems globally (Organization WH, 2006). Billinger et al. identify physical inactivity as a major contributing factor to stroke (Billinger et al., 2014). The mortality rate following a stroke has declined due to interventions like managing cardiovascular risks, smoking cessation, and hypertension prevention programs. Furthermore, studies show that individuals aged 20–59 who engage in regular physical activity have a lower prevalence of stroke. However, physical activity typically diminishes with age (Mozaffarian et al., 2015). Previous research indicates that 77% of stroke survivors lead sedentary lifestyles or have low levels of physical activity (Senes, 2006).

Recent research indicates that regular physical activity aids motor recovery, enhances cardiorespiratory fitness, boosts walking speed and balance, and reduces the risk of recurrent stroke (Mead and Bernhardt, 2011; Saunders et al., 2020). There are strong evidence that there are a bidirectional relationship between participation and higher levels of physical activity in stroke survivors regardless time since stroke (de Diego-Alonso et al., 2024a). Consequently, identifying strategies to enhance participation and adherence in post-stroke physical activity programs is crucial for maintaining and improving physical function and quality of life. Exercise preferences vary across health conditions, such as breast cancer (Rogers et al., 2007; Rogers et al., 2009) and aging (Banks et al., 2012). According to Banks et al., stroke survivors tend to prefer structured group exercises in gyms and community centers (Banks et al., 2012). Moreover, there are studies emphasizing the importance of understanding the needs of stroke survivors when prescribing physical activity and exercise in intervention programs by healthcare professionals, considering the individual perceptions after stroke, the reasons for and against physical activity and exercise, and their impact on daily lives and activities (de Diego-Alonso et al., 2024c). Thus, exercise preferences are influenced by health conditions, living environments, and cultural and social factors (Rogers et al., 2009; Banks et al., 2012).

Validity and reliability studies are essential in developing and adapting measurement tools, as they ensure that the instruments accurately and consistently capture the intended constructs. Such studies are particularly crucial in clinical and rehabilitation settings, where the outcomes directly impact patient care and decision-making (Bazancir-Apaydin and Sari, 2024). To date, there are limited tools available for evaluating exercise preferences and barriers among stroke survivors, and no such scale has been developed or adapted for use in the Turkish language. While some general scales, such as the Exercise Benefits and Barriers Scale (EBBS) (Dilek and Akyol, 2019), have been utilized to assess exercise-related constructs, these tools do not comprehensively address the specific preferences and barriers unique to stroke survivors. The SEPI stands out as a robust tool that integrates both exercise preferences and barriers, providing clinicians and researchers with a more nuanced understanding of this population’s needs. By adapting SEPI to Turkish, this study addresses a critical gap in the literature and provides a valuable resource for clinical and research applications in Turkey.

The Stroke Exercise Preference Inventory (SEPI), originally developed in English, has been used with stroke survivors in Australia (Bonner et al., 2016). Currently, there are no tools available in Turkish literature to assess exercise preferences in stroke patients or other patient groups. The SEPI serves as a useful scale for exploring exercise preferences in individuals who have experienced a stroke, aiding in the development and planning of tailored exercise programs (Bonner et al., 2016). This study aims to validate and analyze the reliability of the Turkish version of SEPI for assessing exercise preferences in stroke patients. The findings highlight the significance of exercise preferences in physiotherapy and rehabilitation practices, with potential for future evaluation in other conditions.

## 2 Methods

### 2.1 Participants

This study was planned as cross-sectional research. The research sample consisted of 99 individuals over the age of 18 referred to Kırıkkale University, Faculty of Health Sciences, Department of Physiotherapy and Rehabilitation, who had a stroke and good cognitive status.

Individuals who had a stroke, had good cognitive status (to have 24 or more from the Mini-Mental Status Assessment), to be able to walk at least 10 min dependently, could speak, read and write Turkish, and volunteered to participate were included in the study. Individuals who were pregnant, could not speak, read or write Turkish, and had any other concomitant neurological and psychiatric disorders were excluded from the study.

The study was carried out in accordance with the Helsinki Declaration. Informed Consent Forms were signed by all participants. This consent allows the use of data collected from individuals. Ethical approval was obtained from the Tokat Gaziosmanpaşa University Clinical Research Ethics Committee (13 April 2023 – decision no.: 83116987–272). The study was registered on ClinicalTrials.gov (NCT05839808).

In the original version of the SEPI, the Intraclass Correlation Coefficient (ICC) value was not calculated. And no other version study has been encountered. Therefore, based on the literature (Portney and Watkins, 2009), the expected reliability level (0.75–0.90) (ρ1 = 0.85), the minimum acceptable reliability level (ρ0 = 0.75) (Andresen, 2000), *α* = 0.05, *β* = 0.20 were taken, and the sample size was determined as 99. The sample size calculation was based on psychometric property evaluations, aligned with Consensus-based Standards for the Selection of Health Measurement Instruments (COSMIN) recommendations. A minimum of 5–10 participants per item was targeted for factor analysis, ensuring sufficient power for reliability and validity assessments (Mokkink et al., 2019). For the 13-item main inventory of the SEPI, including at least 65 individuals was sufficient. The item properties for the SEPI were analyzed among 90 participants as part of the scale development process, while reliability analyses were conducted with 70 participants.

The participants were stable between repeated measurements, as confirmed by their consistent clinical status. The time interval between the test and retest measurements was 7–14 days, ensuring an adequate duration to minimize recall bias while maintaining clinical stability (Arikan et al., 2023). Data collection was conducted by a two rater.

### 2.2 Translation stages

Permission was obtained from the survey developer at the beginning of the study (Bonner et al., 2016). The adaptation process was performed according to the guideline suggested by Beaton et al. (2000).

Forward translation: Two forward translations were obtained by two bilingual translators, one (T1) familiar with the research concept and the other (T2) unaware. The original English version of the SEPI was translated into Turkish.

Synthesis: The resulting translations were checked and turned into a single translation (T12).

Backward translation: The Turkish version created (T12) was translated back into English by two native English-speaking translators who did not know the original version of the questionnaire. Two English back translations (BT1 and BT2) were obtained.

Expert board: The original version of the inventory and all translations (T1, T2, T12, BT1, BT2) were reviewed by the expert committee, and the inventory was finalized. While creating the final version of the inventory, the translations were evaluated in terms of semantic, idiomatic, experiential, and conceptual equivalences.

Prefinal version: The final version created was tested on 30 individuals. Both the meaning features of the items and the answers of the individuals were examined. No unfavorable status was detected.

The expert committee reviewed the translations for semantic, idiomatic, experiential, and conceptual equivalences. Throughout the review process, there was complete agreement among the experts on all items, and no discrepancies were observed. Therefore, statistical analyses to quantify inter-rater reliability, such as the coefficient of variation, were deemed unnecessary.

Content validity was assessed by an expert committee, who evaluated the items for semantic, idiomatic, experiential, and conceptual equivalences. The committee reached full consensus on all items, ensuring that the inventory comprehensively addressed the construct of exercise preferences in stroke survivors.

### 2.3 Instruments

#### 2.3.1 Stroke exercise preference inventory (SEPI)

SEPI, developed in 2016, is a 13-item inventory that also includes 9 items assessing exercise barriers. The SEPI is a useful tool for clinicians to identify the exercise preferences of stroke survivors and to understand their attitudes toward continuing exercise and rehabilitation programs. It facilitates the creation and planning of personalized exercise regimens for individuals who have experienced a stroke. Each item in the SEPI is scored as a percentage. The maximum score for the 13-item exercise preference inventory (SEPI-13) is 1,300, while the minimum score is 0. For the 9-item exercise barriers section, the maximum score is 900, and the minimum is 0. An increasing score in SEPI-13 indicates a higher degree of positive engagement in exercise preferences, whereas an increasing score in the exercise barriers section reflects greater barriers to exercise (Bonner et al., 2016). The SEPI structure consists of seven factors, confirmed via confirmatory factor analysis. While each factor can be calculated individually as the mean of its respective items, the factors are composed as follows:

Factor 1 (Supervision-support): Mean of items Q1 and Q8.

Factor 2 (Confidence-challenge): Mean of items Q2 and Q9.

Factor 3 (Health-wellbeing): Mean of items Q3 and Q10.

Factor 4 (Exercise context): Mean of items Q4 and Q11.

Factor 5 (Home-alone): Mean of items Q5 and Q12.

Factor 6 (Similar others): Mean of items Q6 and Q13.

Factor 7 (Music-TV): Single-item score (Q7).

In this study, however, a total score was calculated by summing all items across factors to provide a comprehensive measure of exercise preferences and barriers. This approach was used for subsequent correlation analyses with external parameters, as it allows for a holistic understanding of participants’ preferences. There is no Turkish version of SEPI. This study was the first to produce a Turkish version.

#### 2.3.2 Mini mental test (MMT)

The MMT was employed to quantitatively evaluate cognitive performance. It comprises 11 items organized under five main categories: orientation, recording memory, attention and calculation, recall, and language, with a total possible score of 30. A score of at least 24 is required. The Turkish version’s validity and reliability were established by Folstein et al. (1975) and Gungen (2002).

#### 2.3.3 Frenchay activities index (FAI)

The FAI is a 15-item index designed to assess the frequency of daily and social activities in individuals with stroke. The initial 10 items request individuals to indicate how often they performed household chores, such as cooking and doing laundry, in the last 3 months. The subsequent five items ask about the frequency of social activities, like traveling and gardening, over the past 6 months. Responses are scored from 0 (never) to 3 (at least once per week), with total scores ranging from 0 (no participation) to 45 (frequent participation) (Schuling et al., 1993). The Turkish version of the FAI has undergone validity and reliability testing (Alaca, 2020).

#### 2.3.4 Stroke-specific quality of life scale (SSQoL)

The SSQoL is a 49-item scale comprising 12 domains designed to evaluate the quality of life in individuals diagnosed with stroke. These domains include energy (3 items), work/productivity (3 items), vision (3 items), personality traits (3 items), family roles (3 items), thinking (3 items), language (5 items), social roles (5 items), self-care (5 items), mood (5 items), upper extremity function (5 items), and mobility (6 items). Each item is rated using a Likert scale ranging from 1 to 5, where a higher score reflects a higher quality of life, and a lower score indicates a lower quality of life (Williams et al., 1999). The Turkish version of the SSQoL has been validated and found reliable (Hakverdioğlu Yönt and Khorshid, 2012).

#### 2.3.5 Exercise benefits/barriers scale (EBBS)

The EBBS consists of 24 items, two open-ended questions, and is organized into six sub-dimensions. Of the 24 items, 12 are statements regarding the benefits of exercise, while the other 12 pertain to barriers to exercise, with negative items being reverse-coded. The scale uses a 4-point Likert rating system, with total scores ranging from 24 to 96. Higher scores reflect a greater perception of exercise benefits and fewer perceived barriers (Sechrist et al., 1987). The Turkish version of the EBBS has been validated and found reliable (Dilek and Akyol, 2019).

#### 2.3.6 Behavioral regulations in exercise questionnaire-2 (BREQ-2)

The BREQ-2 consists of 19 items divided into five subscales: amotivation, external regulation, introjected regulation, identified regulation, and intrinsic regulation. It uses a 5-point Likert scale with scores ranging from 0 to 4 (Mullan et al., 1997). A validity and reliability study has been conducted for the Turkish population (Ersöz et al., 2012).

#### 2.3.7 Functional ambulation category (FAC)

Ambulation level was assessed using the Functional Ambulation Category (FAC), a tool designed to classify walking ability into six categories ranging from 0 (non-functional ambulation) to 5 (independent ambulation on all surfaces). Higher scores indicate better ambulation capacity (Mehrholz et al., 2007).

### 2.4 Statistical analysis

Statistical analysis was performed using the Statistical Package for Social Sciences (SPSS 22.0, SPSS Inc., Chicago, Illinois) for Windows. Confirmatory factor analysis was conducted with Lisrel version 8.80. Statistical data are presented as mean ± standard deviation (X ± SD), median, or percentage (%). The Kolmogorov–Smirnov test was employed to determine whether the data followed a parametric or nonparametric distribution.

Reliability analysis was conducted by testing internal consistency with Cronbach’s alpha and test–retest reliability with the Intraclass Correlation Coefficient (ICC). Cronbach’s *α* values meaning “weak,” “moderate,” “good” and “excellent/strong” are 0–0.69, 0.70–0.79, 0.80–0.89, and 0.90–1.00, respectively. ICC values indicating poor, moderate, good, and excellent reliability are <0.5, 0.5–0.75, 0.75–0.90, and > 0.90, respectively. ICC were calculated using a two-way random effects model ICC (Billinger et al., 2014; Organization WH, 2006) to assess agreement between test–retest measurements. This model accounts for variability across participants and measurements, ensuring a robust evaluation of reliability. To evaluate the agreement and systematic differences between test–retest scores and Bland–Altman plots (95% limits of agreement) were utilized.

Reproducibility was assessed through the standard error of measurement (SEM) and the minimum detectable change (MDC), calculated using the following formulas (Portney, 2020):

MDC_95_: z * SEM * √2, where z = 1.96 (reflecting a 95% confidence) and SEM is the standard error of measurement.

MDC_90_: z * SEM * √2, where z = 1.65 (reflecting a 90% confidence) and SEM is the standard error of measurement.

SEM: SD * √(1-ICC), where SD is the standard deviations of participants, and ICC is the reliability coefficient.

The structural validity of the SEPI was evaluated using both exploratory factor analysis (EFA) and confirmatory factor analysis (CFA). The suitability of the sample tests was evaluated with the Bartlett test, and the sample adequacy was evaluated with the Kaiser Meyer Olkin test (Feise and Menke, 2001). The resulting factor structure was tested with CFA. Fit indices regarding this analysis were also examined (İlhan and Çetin, 2014).

The relationship of SEPI-13 with EBBS, BREQ-2, SSQoLS, and FAI scores was tested with Pearson correlation analysis. Correlation coefficients were interpreted as follows: 0.81–1.00 (excellent), 0.61–0.80 (very good), 0.41–0.60 (good), 0.21–0.40 (weak), and 0.00–0.20 (poor) (Feise and Menke, 2001). To evaluate convergent validity, we hypothesized that SEPI-13 and exercise barriers scores would show strong negative correlations with EBBS and amotivation (BREQ-2), and strong positive correlations with intrinsic motivation (BREQ-2). For divergent validity, we expected SEPI-13 scores to show weak or non-significant correlations with unrelated constructs, such as specific domains of the SSQoLS and FAI.

To assess ceiling and floor effects, the percentages of the minimum and maximum SEPI scores were calculated (Terwee et al., 2007).

Statistical significance value was accepted as *p* < 0.05.

## 3 Results

Although the sample size calculation indicated that 99 participants would be ideal for evaluating psychometric properties, a total of 90 stroke survivors were included in the study. This sample size was determined to be sufficient for conducting validity and reliability analyses based on the results obtained.

The translation process followed the standard forward-backward translation methodology. Two independent bilingual translators performed forward translations of the original SEPI into Turkish, followed by a synthesis of the translations. The synthesized version was then back-translated into English by two native English speakers who were blinded to the original version. An expert committee reviewed the translations for semantic, idiomatic, experiential, and conceptual equivalences, ensuring the Turkish version accurately reflected the original content. The final Turkish version was tested and finalized for validity and reliability.

The expert committee unanimously agreed on the evaluations of semantic, idiomatic, experiential, and conceptual equivalences of the translations. As there were no disagreements among the experts, formal statistical measures of agreement, such as the coefficient of variation, were not performed.

Present study included 90 individuals, 32 female and 58 male, with an average age of 57.56 ± 15.25 years. While the average MMT score of the individuals was 25.77 ± 2.20, the average ambulation level was 3.23 ± 2.41. Other descriptive information about the individuals was presented in Table 1.

**Table 1:** Descriptive characteristics of individuals.

	Test group (*n* = 90)	Retest group (*n* = 70)
	Mean ± SD	
Age	57.56 ± 15.25	55.13 ± 15.90
Weight (kg)	77.37 ± 12.13	77.20 ± 12.06
Length (m)	1.68 ± 0.09	1.69 ± 0.09
BMI (kg/m^2^)	27.40 ± 4.38	27.11 ± 3.96
MMT	25.77 ± 2.20	25.86 ± 2.09
FAC	3.23 ± 2.41	3.29 ± 2.55
Time since stroke (months)	45.91 ± 30.46	44.70 ± 27.65

	*n* (%)	
Gender		
Female	32 (35.6)	23 (32.9)
Male	58 (64.4)	47 (67.1)
Marital status		
Single	16 (17.8)	15 (21.4)
Married	74 (82.8)	55 (78.6)
Education history		
Illiterate	7 (7.8)	5 (7.1)
Primary school	35 (38.9)	23 (32.8)
Middle school	12 (13.3)	9 (12.9)
High school	18 (20.0)	16 (22.9)
Associate degree	4 (4.4)	3 (4.3)
Bachelor degree or above	14 (15.6)	14 (20.0)
Smoking		
Yes	27 (30.0)	21 (30.0)
No	63 (70.0)	49 (70.0)
Alcohol use		
Yes	10 (11.1)	8 (11.4)
No	80 (88.9)	62 (88.6)
Dominant side		
Right	78 (86.7)	61 (87.1)
Left	12 (13.3)	9 (12.9)
Hemiplegic side		
Right	42 (46.7)	33 (47.1)
Left	48 (53.3)	37 (52.9)
Stroke types		
Ischemic	84 (93.3)	66 (94.3)
Hemorrhagic	6 (6.7)	4 (5.7)
Botox application in the last 3 months		
Yes	10 (11.1)	7 (10.0)
No	80 (88.9)	63 (90.0)

BMI, body mass index; MMT, mini mental test; FAC, functional ambulation category.

Content validity of the Turkish version of the SEPI was ensured through evaluations by an expert committee. The experts reached unanimous agreement on all items, confirming that the inventory appropriately addresses the construct of exercise preferences in stroke survivors. The inclusion of participants with preserved good cognitive status aligns with prior studies, enabling accurate comprehension and response to the inventory, and supporting its applicability in regular clinical practice (de Diego-Alonso et al., 2024b).

### 3.1 Reliability

The questionnaire consisted of two sections: SEPI-13 and exercise barriers. During the retest phase, some participants did not complete the exercise barriers section, resulting in a smaller dataset for this analysis (*n* = 53). This was taken into account when interpreting the results.

**Figure 1:**
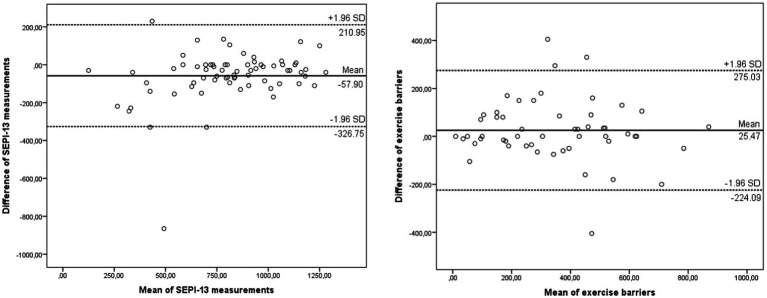
Bland–Altman plots of test and retest scores in SEPI-13 and exercise barriers.

The mean scores for individual items ranged from 47.20 to 82.76%, with corresponding standard deviations between 23.95 and 34.04%. The total score for SEPI-13 had a mean of 824.8 (out of 1,300) with a standard deviation of 278.7. Exercise barriers had a mean score of 342.8% (standard deviation: 204.0%), reflecting the overall scores for the questionnaire. Internal consistency analysis results showed that Cronbach’s *α* was 0.93 for SEPI-13 and 0.90 for exercise barriers. The Cronbach’s alpha values, if each item were deleted, ranged from 0.89 to 0.91, indicating strong internal consistency. ICC values were 0.87 for SEPI-13 and 0.82 for exercise barriers, further supporting the reliability of the scale. The SEM values for SEPI-13 and exercise barriers were 96.9 and 90.0, respectively, while the MDC values were 268.8 and 249.5 within the 95% confidence interval and 226.3 and 210.0 within the 90% confidence interval. These values indicate acceptable measurement precision for both scales (Table 2). The Turkish version of SEPI-13 demonstrated good reliability, with Cronbach’s α values in the excellent range (>0.90) and ICC values between 0.75 and 0.90, classified as good reliability. Bland–Altman plots also demonstrated agreement and consistency between test and retest scores (Figure 1). Bland–Altman plots demonstrated the agreement between test and retest scores. For SEPI-13, the mean difference was 57.90, corresponding to a percentage difference of approximately 4.45% (calculated based on the maximum score of 1,300). For exercise barriers, the mean difference was 25.47, corresponding to a percentage difference of approximately 2.83% (calculated based on the maximum score of 900). These results indicate good agreement and minimal bias between test and retest measurements.

**Table 2:** Test–retest reliability and internal consistency values of the SEPI-13 and exercise barriers (*n* = 70).

	Baseline Mean ± SD	Retest Mean ± SD	Test–retest (ICC_2,1_ and 95% CI)	SEM	MDC_95_	MDC_90_	Internal consistency (Cronbach’s α)
SEPI-13 (*n* = 70)	780.26 ± 289.06	838.16 ± 249.90	0.871 (0.800–0.918)	96.991	268.845	226.323	0.931
Exercise barriers (*n* = 53)	360.47 ± 209.59	335.00 ± 221.45	0.826 (0.716–0.895)	90.033	249.558	210.087	0.904

SEM, standard error measurement; MDC, minimal detectable change; SEPI-13, stroke exercise preference inventory-13.

### 3.2 Validity

**Figure 2:**
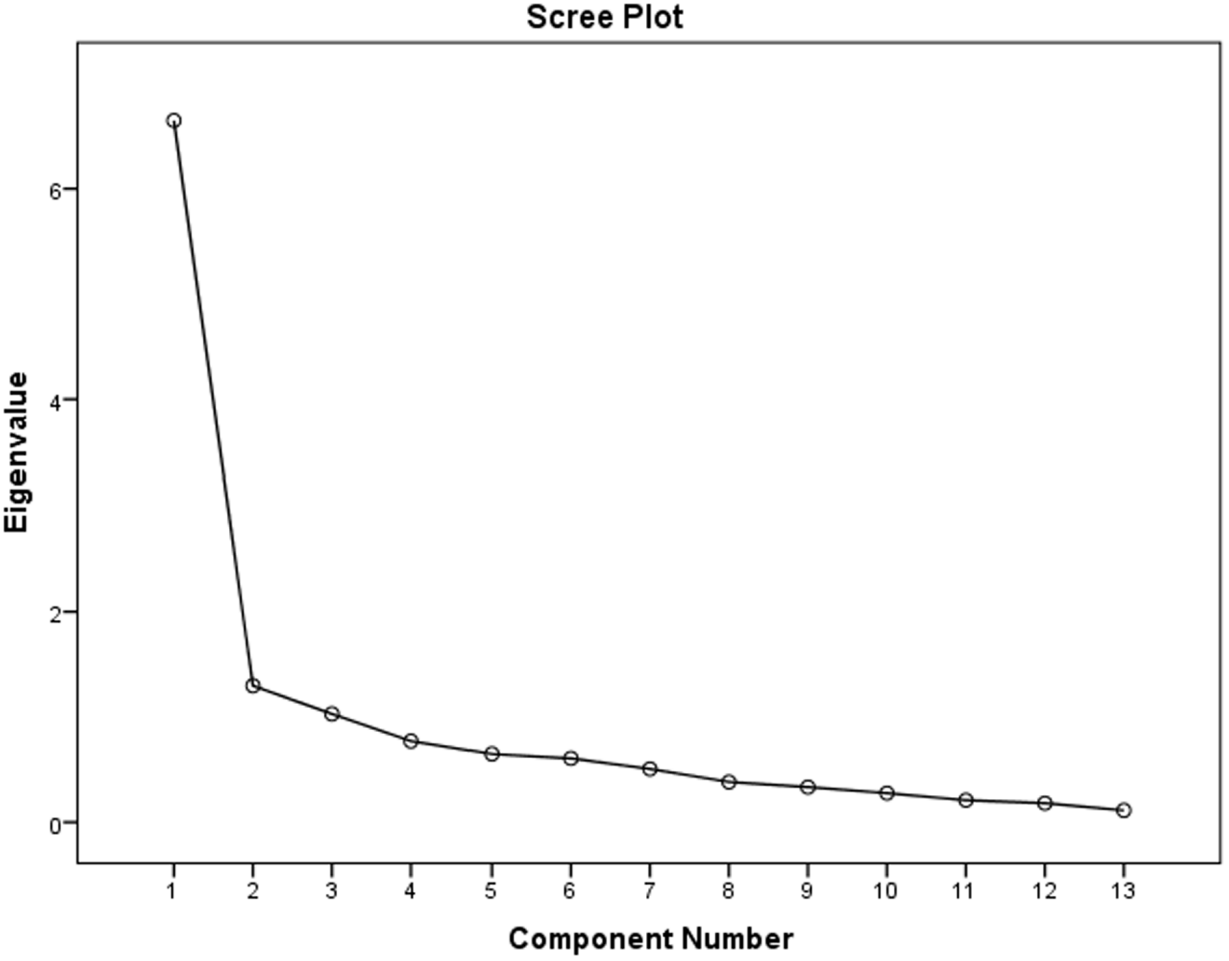
Scree plot of the three-factor structure of SEPI-13.

**Figure 3:**
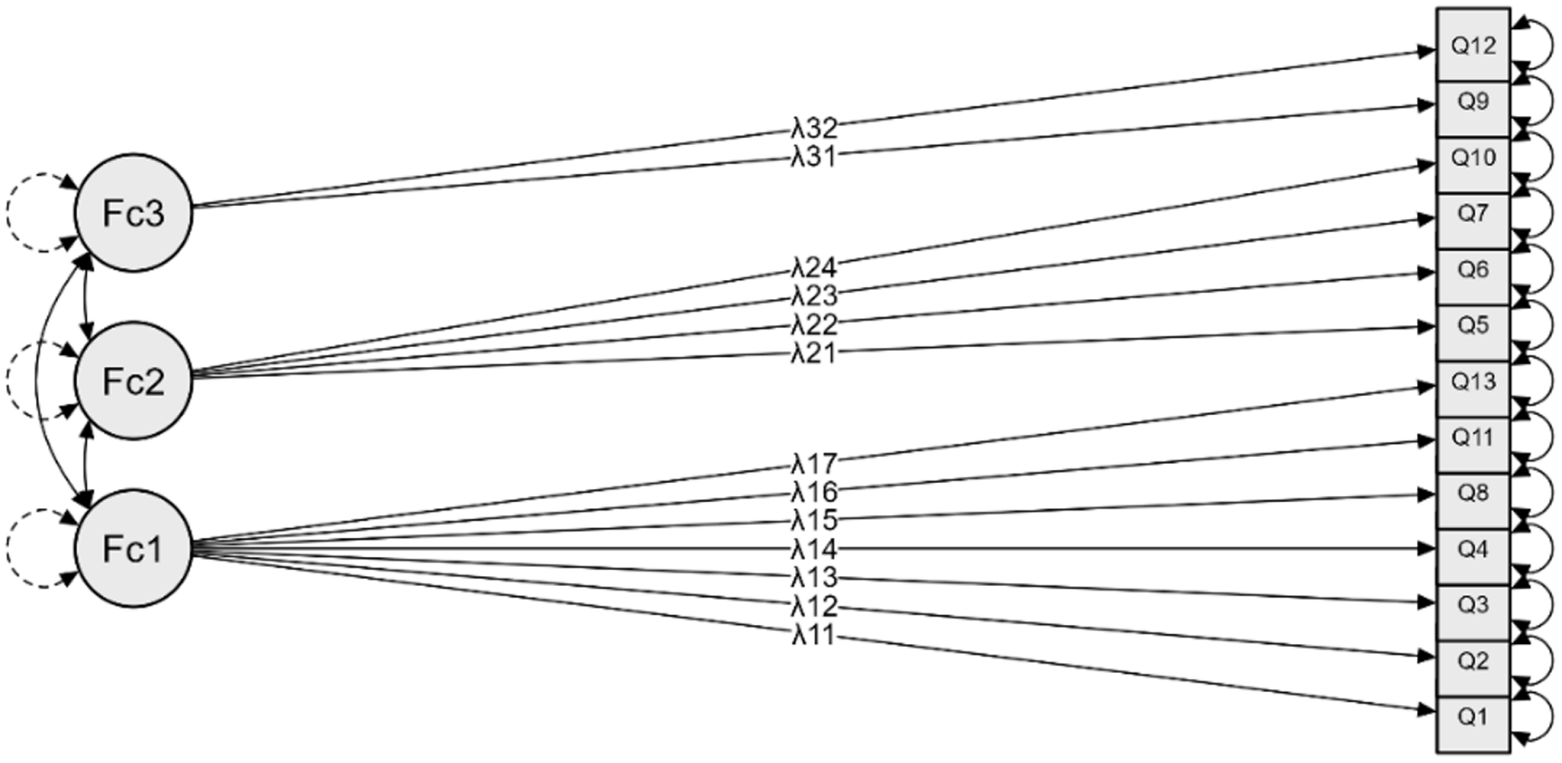
Confirmatory Factor Analysis model plot supporting the three-factor structure of SEPI-13.

The Turkish version of SEPI-13 showed a 3-factor structure (Figure 2). The Kaiser–Meyer–Olkin measure was 0.874. Bartlett’s test of sphericity result was 712.244 (*p* < 0.001). The total variation percentage was found to be 69.029 (Table 3). The 3-factor structure was tested with CFA (Figure 3). The fit indices were largely good (Table 4).

**Table 3:** Three factor structure of SEPI-13 (*n* = 90).

	Factor 1	Factor 2	Factor 3
Q1	0.766		
Q2	0.798		
Q3	0.653		
Q4	0.765		
Q8	0.746		
Q11	0.762		
Q13	0.563		
Q5		0.681	
Q6		0.691	
Q7		0.766	
Q10		0.745	
Q9			0.560
Q12			0.784
Percent variance (%)	32.061	55.508	69.029

SEPI-13, Stroke Exercise Preference Inventory-13.

**Table 4:** Confirmatory Factor Analysis results of the three-factor SEPI-13 model obtained with Principal Component Analysis and Varimax rotation (*n* = 90).

Indices	Good fit measure	Perfect fit measure	Measurement values
CMIN/DF (x^2^/df)	2 < x^2^/df ≤ 3	0 ≤ x^2^/df ≤ 2	(130.570/62) 2.11
SRMR	0.05 < SRMR ≤0.10	0 ≤ SRMR ≤0.05	0.065
RMSEA	0.5 < RMSEA ≤0.10	0 ≤ RMSEA ≤0.05	0.111
IFI	0.90 ≤ IFI < 0.95	0.95 ≤ IFI ≤ 1.00	0.902
CFI	0.90 ≤ CFI < 0.95	0.95 ≤ CFI ≤ 1.00	0.900
GFI	0.90 ≤ GFI < 0.95	0.95 ≤ GFI ≤ 1.00	0.940
NFI	0.90 ≤ NFI < 0.95	0.95 ≤ NFI ≤ 1.00	0.829

CMIN/df, relative Chi-square; SRMR, standardized root mean square residual; RMSEA, root mean square error of approximation; IFI, incremental fit index; CFI, comparative fit index; GFI, goodness of fit index; NFI, normed fit index.

To provide additional context for the correlation results, we have included the mean and standard deviation values for all variables in Table 5. This table offers detailed descriptive statistics to complement the correlations presented in Table 6. The relationship between SEPI-13 and exercise barriers with EBBS, BREQ-2, SSQoLS, and FAI scores was determined for construct validity. For convergent validity, the correlation of SEPI-13 and exercise barriers with EEBS and BREQ-2 was found to be −0.771, 0.719 and 0.732, −0.736, respectively. For divergent validity, SEPI-13 and exercise barriers had insignificant and weak correlations with SSQoLS and FAI, respectively. The correlation of 0.64 between barriers assessed by EBBS and barriers assessed by SEPI indicates that while both scales assess exercise barriers, they may capture different dimensions or levels of barriers. SEPI is designed to explore individual-specific barriers, whereas EBBS captures broader categories of barriers, which may result in a very good correlation rather than a perfect agreement (Table 6). The observed correlations between SEPI-13 and related constructs were consistent with our pre-established hypotheses regarding both magnitude and direction. These findings support the validity of SEPI-13 in assessing exercise preferences and barriers.

**Table 5:** Means and standard deviations of SEPI-13, exercise barriers, EBBS, BREQ-2, SSQoLS, and FAI scores (*n* = 90).

	Mean ± Standard deviations
SEPI-13	824.87 ± 278.74
Exercise barriers	342.83 ± 204.04
EBBS	90.82 ± 17.20
Exercise barriers	30.11 ± 5.74
Exercise benefits	60.66 ± 12.93
BREQ-2	
Intrinsic regulation	17.42 ± 7.05
Introjected regulation	7.53 ± 3.82
External regulation	6.24 ± 3.32
Amotivation	4.10 ± 4.05
SSQoLS	143.76 ± 32.86
Energy	7.80 ± 2.94
Family role	8.22 ± 2.70
Language	17.82 ± 5.19
Mobility	17.21 ± 5.70
Temperament	16.78 ± 21.26
Personality traits	9.01 ± 3.16
Self-care	15.43 ± 5.00
Social care	11.79 ± 5.01
Thinking	8.38 ± 2.93
Upper extremity function	13.82 ± 5.14
Vision	12.08 ± 2.72
Work/ Production	8.32 ± 3.32
FAI	15.39 ± 10.08

SEPI-13, stroke exercise preference inventory-13; EBBS, exercise benefits/ barriers scale; BREQ-2, behavioral regulation in exercise questionnaire; SSQoLS, stroke-specific quality of life scale; FAI, Frenchay activities index.

**Table 6:** SEPI-13 questionnaire correlations with the EBBS, BREQ-2, SSQoLS, and FAI scores (*n* = 90).

	SEPI-13	Exercise barriers
Convergent validity		
EBBS	−0.771**	0.719**
Exercise barriers	−0.602**	0.644**
Exercise benefits	−0.760**	0.670**
BREQ-2		
Intrinsic regulation	0.732**	−0.736**
Introjected regulation	0.476**	−0.337**
External regulation	−0.106	0.338**
Amotivation	−0.541**	0.559**
Divergent validity		
SSQoLS	0.165	−0.266*
Energy	0.154	−0.381**
Family role	0.125	−0.210*
Language	0.077	−0.084
Mobility	0.033	−0.249*
Temperament	−0.035	−0.023
Personality traits	0.250*	−0.262*
Self-care	−0.020	−0.078
Social care	0.149	−0.164
Thinking	0.170	−0.291**
Upper extremity function	−0.018	0.018
Vision	0.130	−0.147
Work/Production	0.147	−0.151
FAI	0.137	−0.300**

SEPI-13, stroke exercise preference inventory-13; EBBS, exercise benefits/barriers scale; BREQ-2, behavioral regulation in exercise questionnaire; SSQoLS, stroke-specific quality of life scale; FAI, Frenchay activities index. **p < 0.01; *p < 0.05.

The questionnaire showed no floor effect and a minimal ceiling effect, with only 2.2% of participants reaching the maximum score. For exercise barriers, the mean score was 342.83 with a standard deviation of 204.04, and the median was 305, ranging from 5 to 890. The percentage of participants who scored the maximum or minimum on the exercise barriers scale was 0%. No floor or ceiling effects were observed for exercise barriers.

## 4 Discussion

In this study, the cultural adaptation, validity, and reliability of the SEPI for individuals with stroke were assessed. The analyses revealed that the Turkish version of the SEPI demonstrated high correlations with the EBBS (exercise barriers and exercise benefits) and intrinsic regulation (BREQ-2), as well as strong internal consistency and test–retest reliability. The translation and cultural adaptation were conducted according to recommended guidelines, with no systematic issues encountered.

According to the test–retest results, the ICC value was found to be 0.87 for SEPI and 0.82 for exercise barriers. As a result of the internal consistency analysis, Cronbach’s *α* value was 0.93 for SEPI and 0.90 for exercise barriers. While the SEM value was 96.99 and 90.03 for SEPI and exercise barriers, respectively, the MDC value was 268.84 and 249.55 for SEPI and exercise barriers, respectively. Additionally, the MDC values at the 90% confidence interval for SEPI-13 and exercise barriers were 226.3 and 210.0, respectively. In line with recent statistical recommendations, the MDC values were reported at both 95 and 90% confidence levels to reflect their utility in different contexts. The MDC at 90% confidence level, which represents a smaller and more clinically achievable change compared to the MDC at 95%, can provide valuable insights for ordinary clinical practice, especially in setting realistic goals and evaluating intervention outcomes. These analyzes were not available in the original version of SEPI (Bonner et al., 2016). At the same time, there are no versions in other languages. However, the results obtained in this study support the high reliability and time-dependent invariance of the Turkish SEPI. Bland Altman plots also consistently supported this conclusion.

The structural validity of the Turkish version of SEPI was assessed through an EFA, resulting in the extraction of a 3-factor structure. This structure was subsequently confirmed using CFA. The Kaiser-Meyer-Olkin sampling adequacy coefficient (0.874) exceeded the recommended threshold (> 0.60) for conducting factor analysis, indicating satisfactory sampling adequacy. In contrast to this study, the original version yielded a 7-factor structure (Bonner et al., 2016). This disparity could be attributed to cultural differences, as perspectives and preferences regarding exercise may vary across populations. However, the confirmation of the factor structure for the Turkish version through CFA demonstrated compatibility between EFA and CFA findings. Consequently, it can be concluded that the Turkish version exhibits structural validity. Our study identified a 3-factor structure for the Turkish version of SEPI, whereas the original study by Bonner et al. (2016) reported a 7-factor structure. This discrepancy may be explained by differences in cultural and linguistic contexts, as well as the methodologies used in factor extraction and rotation. Furthermore, while our sample size (*n* = 90) was smaller than Bonner et al.’s sample (*n* = 134), it still satisfies the recommended criteria for factor analysis, with a sample-to-item ratio of approximately seven. This ratio ensures the robustness and validity of the findings, even with a smaller sample size.

The study assessed the construct validity of SEPI and exercise barriers by examining their relationships with EBBS and BREQ-2 sub-scores for convergent validity, and with FAI and SSQoLS sub-scores for divergent validity. Especially a very good correlation with EBBS and its sub-scores was expected. Likewise, the correlations between the intrinsic regulation and amotivation sub-scores of BREQ-2 and the good and very good level were not surprising. Although these are not the same as SEPI and exercise barriers, they include similar situations. Insignificant and weak correlations with SSQoLS and FAI, which evaluate the quality of life and activities in individuals with stroke, were also expected. Because SEPI, SSQoLS and FAI tools question different situations from each other. In the original version, the relationships of exercise barriers items with disability, depression, anxiety and fatigue were examined (Bonner et al., 2016). Varying degrees of correlation were found from insignificant to good. Although different questionnaires were used than those in the original version, the results obtained were satisfactory in terms of construct validity.

Turkish versions of SEPI and exercise barriers did not show any floor or ceiling effects. This was examined in this study, unlike the original study. This result showed that the survey was sensitive to variations and that the highest and lowest scores did not cause problems in practice.

SEPI is not intended to entirely replace other tools or the compulsory patient interview, but rather to complement them. It provides a structured and quantifiable approach to understanding stroke survivors’ exercise preferences and barriers. While traditional patient interviews allow for in-depth qualitative exploration, SEPI facilitates systematic assessment and comparison across different patient groups, making it a practical addition to clinical practice. By using SEPI alongside interviews, healthcare professionals can obtain both standardized quantitative data and rich qualitative insights, ensuring a comprehensive understanding of patient needs and preferences.

## 5 Limitations

While this study comprehensively explored the psychometric properties of SEPI and exercise barriers, it did not assess responsiveness, which represents a limitation. Future investigations should focus on evaluating the instruments’ ability to detect changes over time or in response to treatment, thereby enhancing their clinical utility and comprehensiveness. While the expert committee demonstrated unanimous agreement on all aspects of the translation review, the absence of statistical measures for inter-rater reliability, such as the coefficient of variation, may be considered a limitation. Future studies could incorporate such analyses to align with standard reporting practices.

## 6 Conclusion

To conclude, we found the SEPI to be a highly reliable clinical tool for evaluating exercise barriers exhibit. The present study demonstrated that the Turkish versions of SEPI and exercise barriers exhibit validity and reliability among Turkish individuals with stroke. It is anticipated that these easy-to-administer and time-saving questionnaire will serve as valuable tools for identifying exercise preferences and barriers among this population, thus addressing a significant gap in the literature.

## Data Availability

The datasets presented in this study can be found in online repositories. The names of the repository/repositories and accession number(s) can be found below: Data supporting the findings can be requested from the corresponding author.
