# Supplementary material for: Cross-cultural adaptation, reliability and validity of the Turkish version of the stroke exercise preference inventory
Source: Front Psychol. 2025 Feb 13;16:1535140. doi: 10.3389/fpsyg.2025.1535140 (PMC11867092; doi:10.3389/fpsyg.2025.1535140)
Supplement: Supplementary file 1 [file Data_Sheet_1.PDF]

# İETE

# İnme Egzersiz Tercih Envanteri

- Bu anket ne tür egzersizleri sevip sevmediğiniz hakkındadır.
- Egzersiz yapmanıza engel olabilecek şeyler hakkında bir bölüm de vardır.
- Cevaplarınız, sizin için en uygun egzersiz türleri hakkında daha fazla bilgi edinmemize yardımcı olacaktır.

---

## A. Egzersiz Tercihleri

Lütfen aşağıdaki ifadelerin her birine ne kadar katıldığınızı belirtiniz:

**Hiç katılmıyorum** **0%** ———— **Tamamen katılıyorum** **100%**

1. Egzersizimin eğitimli bir eğitmen tarafından denetlenmesini severim ☐ %
2. Düzenli bir egzersiz programına katılabileceğimden eminim ☐ %
3. İnme geçirmiş diğer insanlarla egzersiz yapmayı severim ☐ %
4. Kendimi iyi hissettiren egzersizler yapmak benim için önemlidir ☐ %
5. Evde egzersiz yapmayı severim ☐ %
6. Açık havada egzersiz yapmayı severim ☐ %
7. Egzersiz sırasında müzik dinlemeyi veya TV izlemeyi severim ☐ %
8. Egzersizimin nasıl ilerlediğine yönelik geri bildirim almayı severim ☐ %
9. Egzersizleri yaparken zorlanmayı severim ☐ %
10. Benzer yaştaki diğer insanlarla egzersiz yapmayı severim ☐ %
11. Sağlık nedenlerinden dolayı egzersiz yapmayı severim ☐ %
12. Yalnız egzersiz yapmayı severim ☐ %
13. Ailemle veya arkadaşlarımla egzersiz yapmayı severim ☐ %

---

İETE'nin kullanımı ve dağıtımı ücretsizdir.
Alıntı yapma amaçları ve daha fazla ayrıntı için bkz. Bonner ve ark. (2016)

ID ☐

**B. Olası Engeller**

Lütfen aşağıdaki ifadelerin her birine ne kadar katıldığınızı belirtiniz:

| **Hiç katılmıyorum** | | **Tamamen katılıyorum** |
|---|---|---|
| **0%** | ——— | **100%** |

1. Egzersizin başka bir inmeye neden olabileceğinden endişeleniyorum ☐ %
2. Yapmak istediğim egzersiz çok pahalı ☐ %
3. Ağrıya neden olduğu için egzersiz yapmaktan kaçınırım ☐ %
4. Yapmam gereken egzersiz hakkında yeterli bilgiye sahip değilim ☐ %
5. Egzersiz yaparsam düşeceğimden endişeleniyorum ☐ %
6. Egzersiz yapmak istediğim yerlere gitmek zor geliyor ☐ %
7. Güvenli olmadığını düşündüğüm için egzersizden kaçınıyorum ☐ %
8. Egzersiz yapmak için çok yorgun hissediyorum ☐ %
9. Egzersiz yapmak istememe rağmen başlamakta zorlanıyorum ☐ %

İETE'nin kullanımı ve dağıtımı ücretsizdir.
Alıntı yapma amaçları ve daha fazla ayrıntı için bkz. Bonner ve ark. (2016) ID ☐
